# Learning from the pandemic: mortality trends and seasonality of deaths in Australia in 2020

**DOI:** 10.1093/ije/dyac032

**Published:** 2022-03-15

**Authors:** Gabriel Gregory, Lin Zhu, Andrew Hayen, Katy J L Bell

**Affiliations:** School of Public Health, University of Sydney, Sydney, NSW, Australia and; School of Public Health, University of Sydney, Sydney, NSW, Australia and; School of Public Health, University of Technology Sydney, Sydney, NSW, Australia; School of Public Health, University of Sydney, Sydney, NSW, Australia and

**Keywords:** Australia/epidemiology, pandemics, SARS-CoV-2, COVID-19/epidemiology, cause of death/trends, mortality*/trends, interrupted time series

## Abstract

**Aim:**

To assess whether the observed numbers and seasonality of deaths in Australia during 2020 differed from expected trends based on 2015–19 data.

**Methods:**

We used provisional death data from the Australian Bureau of Statistics, stratified by state, age, sex and cause of death. We compared 2020 deaths with 2015-19 deaths using interrupted time series adjusted for time trend and seasonality. We measured the following outcomes along with 95% confidence intervals: observed/expected deaths (rate ratio: RR), change in seasonal variation in mortality (amplitude ratio: AR) and change in week of peak seasonal mortality (phase difference: PD).

**Results:**

Overall 4% fewer deaths from all causes were registered in Australia than expected in 2020 [RR 0·96 (0·95-0·98)] with reductions across states, ages and sex strata. There were fewer deaths from respiratory illness [RR 0·79 (0·76-0·83)] and dementia [RR 0·95 (0·93-0·98)] but more from diabetes [RR 1·08 (1·04-1·13)]. Seasonal variation was reduced for deaths overall [AR 0·94 (0·92-0·95)], and for deaths due to respiratory illnesses [AR 0·78 (0·74-0·83)], dementia [AR 0.92 (0.89-0.95)] and ischaemic heart disease [0.95 (0.90-0.97)].

**Conclusions:**

The observed reductions in respiratory and dementia deaths and the reduced seasonality in ischaemic heart disease deaths may reflect reductions in circulating respiratory (non-SARS-CoV-2) pathogens resulting from the public health measures taken in 2020. The observed increase in diabetes deaths is unexplained and merits further study.

Key Messages

- We undertook an interrupted time series analysis of the number and seasonality of deaths in Australia during 2020 compared with 2015--19.
- We found 4% fewer deaths from all causes during 2020 in Australia than expected, driven by reductions in respiratory and dementia deaths in older age groups.
- There was also reduced seasonality in ischaemic heart disease deaths, and no changes in cerebrovascular or cancer deaths. More Australians died from diabetes than expected in 2020.
- Public health measures adopted in 2020 were associated with incidental mortality reductions in a number of non- COVID diseases. Retaining some of the adopted behavioural changes in the post-pandemic era merits careful consideration.

## Introduction

There were increased deaths in 2020 associated with the COVID-19 pandemic in many countries, including Germany, France, Italy, Switzerland, Portugal, Sweden, Russia, Brazil, Peru, Israel, Japan, the UK, the USA and Europe as a whole.^1^^,^^2^ Much of the excess mortality was directly attributed to COVID-19,^3^ with a further portion attributed to undiagnosed COVID-19.^4^^,^^5^ For non-COVID disease-specific deaths, both increases and decreases were observed. For example in the USA there were increases in deaths from cardiovascular disease, Alzheimer’s disease and pneumonia (a proportion of which may have had undiagnosed COVID-19), and decreases in deaths from chronic lower respiratory disease.^6^ In Northern Italy during the first 6 months of the pandemic, there were increased deaths from cardiovascular disease and diabetes and decreased deaths from cancer,^7^ and the UK reported increases in deaths from cardiovascular disease and stroke during the earlier months of 2020.^8^ Drug overdose deaths increased in the USA in 2020^6^ with some evidence of peaks associated with the announcement of a public health emergency and rises in unemployment,^9^ whereas data from 21 countries found no change or in some instances declines in the observed number of suicides in 2020.^10^

In contrast to the above, mortality rates have tended not to increase in countries where there was successful suppression (and often temporary elimination) of SARS-CoV-2 infection. For example, New Zealand^11^ and Denmark^12^ both observed decreases in overall mortality for the year. A large Bayesian analysis of mortality rates in 21 industrialized countries, through to May 2020, identified six countries with a likely overall decline in total mortality including Australia, New Zealand and four European countries.^13^ In New Zealand it was hypothesized that the reduction was due to the absence of an influenza epidemic,^11^ which may also have occurred in Australia.^14^ However, the pandemic and associated restrictions may have affected total mortality in a more complex way,^15–17^ with possible changes in the number of deaths due to causes other than directly attributable to influenza. Australia’s pandemic response resulted in a much lower COVID-19 and excess death rate in 2020 compared with many other countries,^18^ and access to health care was relatively preserved during the year.

The overall objective of this study was to assess the potential impacts of the Australian pandemic response on non-COVID-19 disease mortality. Our primary aim was to compare the expected with the observed number of deaths in Australia in 2020 using available provisional mortality data, stratified by cause of death as well as by state, age and sex. Our secondary aim was to explore a hypothesis that in addition to a change in the total number of deaths, the timing of deaths throughout the year (‘seasonality’) may have changed. Assessment of the seasonal pattern may allow detection of more subtle changes in mortality during 2020. The nature of some seasonality measures as ratios (see Methods below) means that these outcomes are less confounded by the possibility of incomplete reporting of deaths in 2020. Moreover, changes in seasonal pattern can provide some evidence that any observed reductions are related to a reduction in factors that drive the seasonality of disease, such as reductions in circulating respiratory viruses.

## Methods

### Data inputs

Data for this study were sourced from a publicly available dataset maintained by the Australian Bureau of Statistics (ABS).^19^ Mortality data were available stratified by age and sex, or by state, or by cause, but not by combinations of these. At the time the study was conducted, provisional data were available for the number of doctor-certified deaths each week in Australia until December 2020 (data used were those released by ABS on 24 November 2021). The data are considered provisional in that reported numbers and causes of death remain subject to change as additional data are received. A death is considered doctor-certified when a Medical Certificate of Cause of Death has been written by a medical practitioner. In Australia, all deaths are doctor-certified except when they occur in circumstances requiring referral to the coroner. Deaths due to unknown or external causes (e.g. accidents, suicides, assaults) are generally referred (∼10-15% of deaths), although precise criteria vary between states. The Australian Medical Certificate of Cause of Death is based on that recommended by the World Health Organization (WHO) for international use.^20^ This asks the medical practitioner to record the sequence of events leading to death, with the proximate cause at the top (e.g. Acute Respiratory Distress Syndrome), and each antecedent causes listed below (e.g. pneumonia), with the underlying cause listed at the bottom (e.g. COVID-19).^21^ After registration of the death, the information is passed on to the ABS where staff code the causes of death, according to the WHO’s International Statistical Classification of Diseases and Related Health Problems-10th Revision (ICD-10).^20^ The underlying cause of death is used for cause-specific mortality data.

Data were available as weekly totals of deaths, grouped by the date on which the death occurred. Week 1 of a given year begins on 1 January, and each of Weeks 1-52 contain 7 days. Week 53 contains 1 day (31 December) or 2 days during leap years. Published causes of death were grouped according to the International Classification of Diseases (ICD-10) and are limited to: COVID-19 (U07), ischaemic heart disease (I20-25), cerebrovascular disease (I60-69), respiratory disease (J00-J99), chronic lower respiratory disease (J40-47), influenza (J09-J11), pneumonia (J12-18), cancer (C00-C97, D45, D46, D47.1 or D47.3-D47.5), diabetes (E10-14), dementia (F01, F03 or G30).

### Data analysis

We assessed for changes in mortality in 2020 in each of the above categories (demographic group, state, cause of death) using interrupted time series^22^ adjusted to account for time trends and seasonality. Given the over-dispersed weekly death count data, models were fitted by quasi-Poisson regression.^23^ Time was modelled as a linear predictor and seasonality was accounted for with harmonic terms (pairs of sine and cosines). The interruption was modelled by including an indicator variable for 2020 (‘the effect of 2020’) and allowing this variable to interact with the linear predictor. The general formula for the starting model including all terms was: weekly death ∼ time trend + seasonal trend + Covid indicator + interaction term (seasonal trend × Covid indicator). Separate models were fitted for each state and territory, sex and age strata and cause of death. Interaction terms were included in models where their *P*-value was <0.05. We verified the chosen models by checking the autocorrelation and partial autocorrelation of residuals. Analyses were performed in R 3.6.3.^24^

### Outcome measures

Change in mortality in 2020 was assessed by the ratio of observed deaths to expected deaths. The observed number of deaths refers to the total number of doctor-certified deaths that took place in Weeks 1-52 of 2020. To calculate the expected (counterfactual) number of deaths, we forecast the number of deaths in 2020 using the selected model but with the effect of 2020 set to zero. We calculated the ratio of observed to expected deaths and produced confidence intervals using 95% confidence limits of our counterfactual projections.

Changes to seasonality in 2020 were primarily assessed by changes in the extent of seasonal variation [we also examined the timing of the seasonal peak ‘phase’ and ‘phase difference’ (PD), see Supplementary Material, available as Supplementary data at* IJE* online, for more details]. The amplitude was defined to be the maximum contribution of the seasonal terms, expressed as a rate ratio.^25^ For example, an amplitude of 1.20 indicates 20% more deaths in the week of peak mortality than if there were no seasonal pattern to the deaths. Finally, an amplitude ratio (AR) was calculated by dividing the amplitude fitted for 2020 by the amplitude fitted for 2015-19. These outcome measures were produced via transformations of the regression coefficients for the harmonic terms of the fitted model and approximate 95% confidence intervals were produced using the delta method.^26^

## Results

### Overall mortality

**Figure 1:**
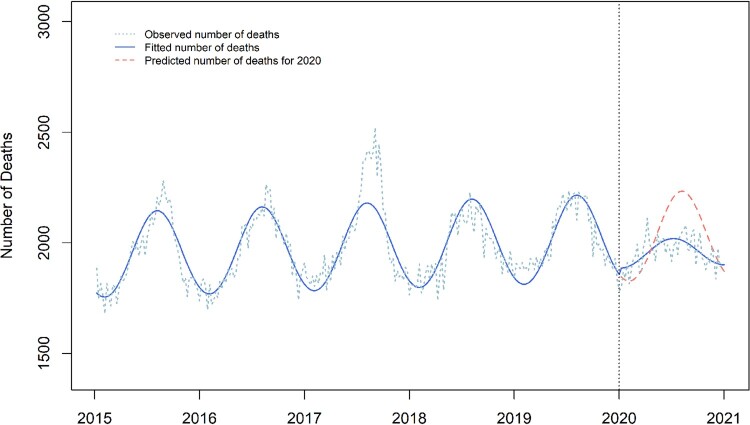
Expected vs observed all-cause mortality (doctor-certified deaths) in Australia 2015–20. This graph compares the observed number of doctor-certified deaths each week in Australia from 2015–20 (blue dashed line) with the number of deaths fitted by the optimal generalized linear model (blue solid line). In 2020, the counterfactual prediction of the fitted model is additionally presented (red dashed line). Note the significant reduction in the amplitude between the two fitted curves, demonstrating the observed reduction in seasonality in 2020

In Weeks 1 to 52 of 2020, there were proportionately 4% fewer doctor-reported deaths than projected (RR 0.96, 95% CI 0.95, 0.98; Figure 1, Table 1). The seasonal amplitude was reduced from 1·10 during 2015-19 to 1·03 in 2020 (amplitude ratio 0·94; 95% CI 0·92, 0·95). The week of peak modelled mortality occurred 5 weeks earlier than in 2015-19 (95% CI 1, 8 weeks, Supplementary Appendix Table 3, available as Supplementary data at *IJE* online).

**Table 1:** Certified deaths in each Australian state and territory and in Australia overall from 1 January 2020 to 29 December 2020

State	Expected deaths	Observed deaths	Absolute reductions	Ratio (95% CI)	Amplitude (2015-19)	Amplitude (2020)	Ratio (95% CI)
NSW	48 829	47 931	898	0.98 (0.96, 1.00)	1.13	1.05	0.93 (0.91, 0.95)
Victoria	34 599	34 230	369	0.99 (0.97, 1.00)	1.10	1.07	0.98 (0.96, 1.00)
Queensland	30 736	29 306	1430	0.95 (0.93, 0.97)	1.08	1.03	0.93 (0.92, 0.97)
South Australia	11 275	11 106	169	0.98 (0.96, 1.01)	1.10	1.04	0.93 (0.90, 0.97)
Western Australia	12 496	12 267	229	0.98 (0.96, 1.00)	1.09	1.04	0.95 (0.92, 0.98)
Tasmania	3803	3700	103	0.97 (0.93, 1.01)	1.12	1.06	0.94 (0.90, 0.99)
Northern Territory + ACT	2925	2906	19	0.99 (0.95, 1.04)	1.09	1.05	1.0 (NA^a^)
Australia	105 463	101 708	3755	0.96 (0.95, 0.98)	1.10	1.03	0.94 (0.92, 0.95)

NSW, New South Wales; ACT, Australian Capital Territories; NA, not available.

a Fitted model contained no seasonal interaction.

**Table 3:** Certified deaths by cause of death in Australia from 1 January 2020 to 29 December 2020

Cause	Expected deaths^a^	Observed deaths^a^	Absolute reductions	Ratio (95% CI)	Amplitude (2015-19)	Amplitude (2020)	Ratio (95% CI)
Total respiratory	15 207	12 055	3152	0.79 (0.76, 0.83)	1.29	1.05	0.78 (0.74, 0.83)
Influenza and pneumonia	3848	2122	1698	0.56 (0.51, 0.61)	1.50	1.25	0.78 (0.68, 0.91)
Pneumonia	2903	2105	798	0.73 (0.69, 0.77)	1.33	1.23	0.91 (0.84, 0.98)
Chronic respiratory	7726	6704	1022	0.87 (0.84, 0.90)	1.24	1.00	0.79 (0.75, 0.83)
Cancer	48 219	47 800	419	0.99 (0.98, 1.00)	1.01	1.00	1.0 (NA^b^)
Ischaemic heart disease	13 577	13 513	64	1.00 (0.97, 1.02)	1.15	1.09	0.95 (0.92, 0.97)
Cerebrovascular disease	8992	8971	21	1.00 (0.97, 1.02)	1.11	1.09	1.0 (NA^b^)
Dementia	15 172	14 463	709	0.95 (0.93, 0.98)	1.14	1.05	0.92 (0.89, 0.95)
Diabetes	4523	4906	−383	1.08 (1.04, 1.13)	1.12	1.12	1.0 (NA^b^)
All causes^c^	105 463	101 708	3755	0.96 (0.95, 0.98)	1.10	1.03	0.94 (0.92, 0.95)

NA, not available.

a Through to 29 December 2020.

b Fitted model contained no seasonal interaction.

c Influenza and pneumonia, Pneumonia and Chronic Respiratory are included in Total Respiratory counts. All causes also include COVID-19 deaths.

Fewer than expected (all-cause) deaths were recorded in all states and territories (Table 1), with the greatest proportional and absolute reduction occurring in Queensland (RR 0·95, 95% CI 0·93, 0·97). These reductions were all associated with reductions in seasonal amplitude (ARs between 0·93 and 0·98) in the states but not the territories (Northern Territory and Australian Capital Territories; AR 1·0). The larger reduction in Queensland arises from a temporal trend for increasing number of deaths across 2015–19 which resulted in a higher expected number of deaths for 2020 (Supplementary Appendix Figure 1, available as Supplementary data at *IJE* online).

A consistent pattern of fewer than expected deaths (all-causes) was observed in all strata of age and sex (Table 2; RRs between 0·94 and 1.00). These reductions were accompanied by reductions in seasonal amplitude in males aged over 64 years and in females aged over 44 years, with the reduction in seasonal amplitude being greater in each successively older age bracket for females. No change in seasonal amplitude was detected in males aged 0-64 or in females aged 0-44.

**Table 2:** Certified deaths by age and sex in Australia from 1 January 2020 to 29 December 2020

Sex	Age	Expected deaths^a^	Observed deaths^a^	Absolute reductions	Ratio (95% CI)	Amplitude (2015-19)	Amplitude (2020)	Ratio (95% CI)
Male	0**-**44	1569	1474	95	0.94 (0.88, 1.00)	1.02	1.02	1.0 (NA^b^)
	45**-**64	8683	8614	69	0.99 (0.97, 1.02)	1.04	1.04	1.0 (NA^b^)
	65**-**74	14 020	13 737	283	0.98 (0.96, 1.00)	1.07	1.03	0.94 (0.96, 0.99)
	75**-**84	21 930	21 793	137	0.99 (0.98, 1.01)	1.10	1.04	0.95 (0.93, 0.97)
	85+	26 623	25 725	898	0.97 (0.95, 0.99)	1.15	1.09	0.94 (0.92, 0.97)
Female	0**-**44	1349	1295	54	0.96 (0.90, 1.02)	1.04	1.04	1.0 (NA^b^)
	45**-**64	6413	6357	56	0.99 (0.96, 1.02)	1.04	1.02	0.98 (0.95, 1.02)
	65**-**74	9553	9247	306	0.97 (0.94, 0.99)	1.06	1.03	0.97 (0.94, 1.00)
	75**-**84	17 215	17 206	9	1.00 (0.98, 1.02)	1.10	1.04	0.94 (0.92, 0.97)
	85+	37 299	35 995	1304	0.97 (0.95, 0.98)	1.14	1.06	0.92 (0.90, 0.94)

NA, not available.

a Through to 29 December 2020.

b Fitted model contained no seasonal interaction.

### Cause-specific mortality

**Figure 2:**
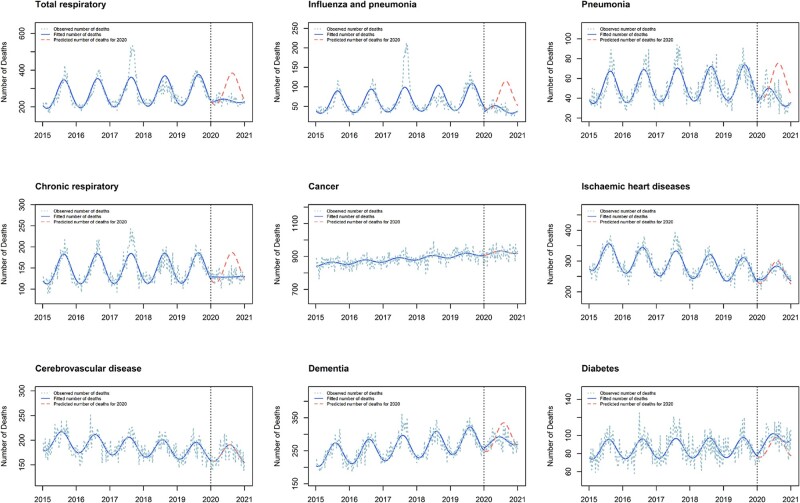
Expected vs observed mortality by cause of death Australia 2015–20. This graph compares the observed number of doctor-certified deaths each week in Australia from 2015–20 (blue dashed line) with the number of deaths fitted by the optimal generalized linear model (blue solid line) for nine causes of death (total respiratory, influenza and pneumonia, pneumonia, chronic respiratory, cancer, ischaemic heart disease, cerebrovascular disease, dementia, diabetes). In 2020, the counterfactual prediction of the fitted model is additionally presented (red dashed line). Note the significant reduction in the amplitude between the two fitted curves, demonstrating the observed reduction in seasonality in 2020

There were fewer reported deaths than expected for total respiratory deaths (RR 0·79, 95% CI 0·76, 0·83), including deaths from influenza and pneumonia (RR 0·56, 95% CI 0·51, 0·61), pneumonia (RR 0·73, 95% CI 0·69, 0·77) and chronic respiratory disease (RR 0·87, 95% CI 0·84, 0·90) (Figure 2, Table 3). There were also fewer deaths than expected for dementia (RR 0·95, 95% CI 0·93, 0·98). These reductions were accompanied by reductions in seasonal amplitude (ARs 0·78- 0·92). Although there was no evidence of a reduction in number of deaths from ischaemic heart disease (RR 1.00, 95% CI 0.97, 1.02), we did observe a reduction in seasonal amplitude (AR 0.95, 95% CI 0.92, 0.97). There were no changes in number or seasonality of deaths from cancer or cerebrovascular disease (RR 1·00, AR 1·00 for both). Finally, the number of deaths from diabetes was greater than expected (RR 1·08, 95% CI 1·04, 1·13) with no evidence of a change in amplitude (AR 1.00) or phase (PD -3, 95% CI -8, 1).

## Discussion

We found that 4% fewer deaths than expected were medically registered in Australia in 2020, with consistent reductions across state, age and sex strata. There was also consistent decreased seasonal variation in overall mortality (from certified causes) across states and territories, with more pronounced reductions in older age brackets. We found strong evidence of reduced mortality (number and seasonality) from respiratory illness and dementia, and some evidence of reduced mortality (seasonality only) from ischaemic heart disease. There was no evidence of a change in mortality from cerebrovascular disease or cancer, and strong evidence of increased deaths from diabetes.

Three other studies have reported on the number of deaths in Australia in 2020. The Australian Bureau of Statistics (ABS) released a report that estimated excess mortality through to August 2020, using a cyclical linear regression model with robust estimators.^27^ The Australian Actuaries Institute published a report that estimated excess mortality to December 2020, using analysis methods that allowed for later reported deaths and changes in the size and age structure of the Australian population.^28^ Most recently, an analysis of change in life expectancy in Australia in 2020 using a life-table method was published.^29^ Our study’s findings agree with those of the other reports, and it adds value by presenting potential impacts of the Australian pandemic response on both total and seasonal components of changes in mortality in 2020.

The overall reduction in total certified deaths is consistent with prior reports of reduced mortality in Australia^13^ and adds to evidence from New Zealand^11^ and Scandinavia^30^^,^^31^ suggesting that some societal changes may have saved lives from non-COVID causes beyond controlling the pandemic. The reduction in respiratory deaths is consistent with known reductions in influenza admissions in Australia^14^ and internationally noted reductions in deaths from chronic respiratory disease^6^ and asthma exacerbations.^32^ The reduction in seasonality in ischaemic heart disease deaths is also in keeping with the established causative role of influenza.^33^ Our findings further suggest substantial mortality benefits from reductions in other circulating respiratory pathogens, coincidentally reduced by the pandemic control measures. The full extent of the health benefits from these non-pharmaceutical interventions in preventing both respiratory and ischaemic heart disease deaths may have been underappreciated before the pandemic.

The observed reduction in total deaths due to dementia in Australia contrasts with international findings,^6^^,^^34^ where increased dementia deaths were found in regions overwhelmed by COVID-19,^35^ with many occurring outside hospital.^36^ Deaths in those countries may have been due to undiagnosed coronavirus infections,^37^ with dementia recorded as the underlying cause and an unknown respiratory pathogen as the proximate cause. Similarly in Australia, many deaths of the people with known dementia in 2015–19 may have had dementia recorded as the underlying cause of death, with respiratory pathogens recognized as the proximate cause. This would then result in an observed reduction in dementia deaths with the reduced circulation of respiratory pathogens in 2020.

The absence of increases in total coronary heart disease and cerebrovascular deaths contrasts with increases noted in the USA,^6^ UK^8^ and Italy.^7^ Considering ways in which the Australian experience differed from the international experience (e.g. relatively preserved access to and use of health care services^38^), our finding suggests that observed increases elsewhere may relate to health system overload and changes in health-seeking behaviour, as well as under-ascertainment of COVID-19 deaths.^37^ Reductions in primary care contacts for cardiovascular disease and diabetes were observed in the UK^39^ and in emergency department presentations across a wide range of severities in Italy.^7^

There were increased total deaths from diabetes in Australia as were observed in Italy,^7^ and Norway during the first wave.^40^ In the latter report, which focuses on the effects of lockdown (there were 216 COVID-19 deaths during the study period), the authors speculated that the observed 45% increase in diabetes deaths may have been due to some patients with diabetes inappropriately avoiding health care. This explanation that may also apply to the Australian findings, a hypothesis supported by data showing increases in diabetes deaths coincident with the two periods of stay-at-home orders (lockdowns) in the state of Victoria in 2020.^41^ An alternative hypothesis is that the excess diabetes deaths could represent undetected COVID-19 deaths.^28^ Further research into the observed increase in diabetes deaths in Australia and elsewhere may yield insights into these and other hypotheses.

There are a number of limitations to our study. We excluded deaths referred to a coroner, including suicides, road traffic accidents and sudden deaths (many of which are due to ischaemic heart disease). The ABS recently released a report on the leading causes of death for 2020 which includes both doctor- and coroner-certified deaths, with findings in keeping with those of the current analysis (seasonal trends were not examined in that report).^42^ In addition, they reported decreases in deaths due to both suicides and road traffic accidents in Australia in 2020. The data in the ABS report indicate that the proportion of overall deaths referred to the coroner (approximately 11%) and of ischaemic heart disease deaths (approximately 18%) did not change for 2020 compared with previous years, supporting the validity of our findings for doctor-certified deaths.

We did not account for competing risks in our analysis of cause-specific deaths, specifically that a decrease in respiratory deaths may result in increased deaths from non-respiratory causes, making our estimates for reductions in non-respiratory deaths conservative (and our estimate for increase in diabetes deaths an overestimate). We also did not account for the time-changing age profile of the population and the slowing in population growth (due to no overseas migration during the pandemic).^43^ It will be important to calculate age-standardized mortality rates once new 2021 Census data become available to enumerate the underlying Australian population. However, it seems unlikely that a changed population structure in 2020 would account for our findings. We anticipate that the main change in the population structure would be fewer young adults than before the pandemic (due to border restrictions preventing entry of international students), with relative preservation of the older age groups in the Australian population. The Australian Actuaries Institute report allowed for estimated changes in the size and age structure of the Australian population, and had similar findings to our analysis.^28^

We were unable to combine stratifications by state, age, sex and cause to explore whether differences in overall mortality between states or age groups might relate to differences in causes of death. Finally, although the data released by the ABS in November 2021 are of high quality, they remains provisional. ABS records suggest 97% of deaths are recorded within 3 months of occurrence and over 99% by 5 months,^42^ suggesting minimal (<1%) impact of incomplete reporting on key measures in this study.

Australia was one of few countries to experience significant suppression of COVID-19 spread in 2020, through a complex intervention consisting of travel restrictions, testing, tracing and isolation, periods of stay at home public health orders, and quarantine requirements. Other contributing factors to the successful response include being an island nation with a relatively small and geographically dispersed population. There have also been adoption of a number of behavioural changes including staying home from work (and school) if symptomatic with a respiratory infection, improved hygiene through hand washing and sanitizer, and increased use of face masks (mandated indoors and on public transport during outbreaks in 2020). By exploring cause of death data, this study contributes to a growing understanding of the causal pathways explaining observed mortality reductions associated with successful suppression, which may be attributable to reductions in the number of circulating respiratory pathogens.

Although international variation in experience with the COVID-19 pandemic constitutes a ‘natural experiment’, this study out of necessity compares the effect of a composite and time-varying intervention with other complex interventions imposed in different populations worldwide. Further, whereas mortality was reduced overall in Australia, this benefit may not have applied equally to underserved subgroups. Additional studies are required to explore the effects of the 2020 pandemic and associated societal changes. Further research could investigate changes in morbidity and mortality from a wider range of illnesses and populations and explore the long-term effects of these sudden societal and behavioural changes whose final impact remains to be seen. Longer term studies may also explore the impacts of delayed health care such as cancer screening.^37^

## Conclusions

In conclusion, public health measures adopted in Australia during 2020 were associated with an overall reduction in all-cause mortality, due to reductions in a number of non-COVID diseases. Our results support the need for increased uptake of existing interventions to prevent respiratory infections, including vaccination programmes, and suggest potential benefits of retaining some of the behavioural (e.g. improved hygiene, staying at home when symptomatic) and policy (e.g. flexibility to work from home where possible, improved ventilation of workplaces and schools) changes, in the long term.

## Ethics approval

In this negligible risk research study, we analysed publicly available datasets of non-identifiable aggregated data, and our investigation was therefore exempt from formal ethics review.

## Data availability

The data underlying this article are available in the Australian Bureau of Statistics Provisional Mortality Statistics, at [https://www.abs.gov.au/statistics/health/causes-death/provisional-mortality-statistics].

## Supplementary data

Supplementary data are available at *IJE* online.

## Author contributions

G.G. contributed to conceptualization, did the data analysis, contributed to data interpretation and wrote the original draft of the manuscript. L.Z. did the data analysis, contributed to data interpretation, contributed to supervision and reviewed and edited the manuscript. A.H. contributed to data interpretation, contributed to supervision and reviewed and edited the manuscript. K.B. conceived of the study, contributed to data interpretation, was primary supervisor and reviewed and edited the manuscript.

## Funding

There was no specific funding for the study. K.B. is supported by an Australian National Health and Medical Research Council (NHMRC) Investigator Grant (#1174523).

## Conflict of interest

None declared.

## Supplementary Material

dyac032_Supplementary_Data
