# Supplementary material for: Learning from the pandemic: mortality trends and seasonality of deaths in Australia in 2020
Source: Int J Epidemiol. 2022 Mar 15;51(3):718–26. doi: 10.1093/ije/dyac032 (PMC9189967; doi:10.1093/ije/dyac032)
Supplement: dyac032_Supplementary_Data [file dyac032_supplementary_data.docx]

**Supplementary Material Part 1: Seasonality outcomes**

Generalised linear models were fitted such that the full model was of the form:

log(E[Y_t_]) = (a_0_+a_1_·t’) + (b_0_+b_1_·t’) ·t + (c_0_+c_1_·t’) ·cos(t·2π/52) + (d_0_+d_1_·t’) ·sin(t·2π/52)

where t is the week index (where week 1 of 2015 is considered week 1, etc.),

and Y_t_ is the number of deaths in week t,

and t’ is an indicator variable taking on the value 0 in 2015-19 and 1 in 2020 (i.e. t’ = I(t>260))

and a_0_, a_1,_ b_0_, b_1,_ c_0_, c_1,_ d_0_, d_1_ are the fitted coefficients (which are possibly zero if the full model was not chosen).

The above model reduces to the form:

log(E[Y_t_]) = A + B·t + C·cos(t·2π/52) + D·cos(t·2π/52),

for some constants A, B, C, D when t is restricted to either the weeks of 2015-19 or to the weeks of 2020.

Note that

C·cos(t·2π/52) + D·sin(t·2π/52) = F·cos((t-φ) ·2π/52),

where F = sqrt(C^2^ + B^2^) ,

and φ can be determined by applying the inverse tangent function to D/C and making appropriate modifications depending on the quadrant in which (C, D) lies.

Hence the model restricted to a particular time-period 2015-19 or 2020 is of the form

log(E[Y_t_]) = A + B·t + F·cos((t-φ) ·2π/52), i.e.

E[Y_t_] =K · T(t) · S(t),

where K = exp(A) is some constant factor, T(t) = exp(Bt) is a multiplicative factor related to the overall trend, and S(t) = exp(F · cos((t-φ) ·2π/52)) is a multiplicative factor related to seasonality.

S(t) is maximal when t = φ, which we define to be the “phase” of the seasonal component and attains maximal value G=exp(F), which we define to be the “amplitude” of the seasonal component.

The 2015-19 amplitude G_0_ and phase φ_0_ are determined from the above calculations with A=a_0_, B=b_0_, C=c_0_, D=d_0_. Similarly the 2020 amplitude G_1_ and phase φ_1_ are determined from the above procedure with A=a_0_+a_1_, B=b_0_+b_1_, C=c_0_+c_1_, D=d_0_+d_1_.

The amplitude ratio (AR) is then calculated as G_1_/G_0_ and the phase difference is φ_1_ - φ_0_ restricted to the interval (-26, 26] by addition or subtraction of 52 as necessary.

**Supplementary Material Part 2: Changes in the timing of the seasonal peak (‘phase’) in 2020 compared to 2015-2019**

In addition to changes in the ‘amplitude’ or extent of seasonal variation reported in the main paper, we also assessed changes in the ‘phase’ or timing of the seasonal peak which we report here. The phase was defined to be the week in which the contribution of the seasonal terms was greatest, i.e. the week in which the number of deaths fitted by the chosen model was most in excess of the overall linear trend. The phase difference (PD), measured in weeks, was calculated by subtracting the phase fitted for 2015-19 from the phase fitted for 2020. These outcome measures were produced via transformations of the regression coefficients for the harmonic terms of the fitted model and approximate 95% confidence intervals were produced using the delta method.^1^

**Overall mortality**

The week of peak modelled mortality occurred 5 weeks earlier than in 2015-19 (95% CI 1-8 weeks). Point estimates were suggestive of peak seasonal mortality occurring 1-6 weeks earlier in many of the larger states, and in most age groups. The modelled peak in seasonal mortality occurred earlier than expected among females aged 45-64 (PD -16, 95% CI -29 to -3) and 65-74 (PD -12, 95% CI -20 to -4) as well as among males aged 0-44 (PD -16, 95% CI -26 to -7).

**Cause-specific mortality**

For all respiratory illnesses studied (chronic respiratory disease, influenza and pneumonia, and all respiratory causes), as well as for dementia there was a change in phase of approximately 3-4 months from the usual peak in mid-August (week 33) to peaks in May (weeks 17-22). There was no change in phase in deaths from ischaemic heart disease, cerebrovascular disease, cancer, or diabetes.

**References**

1. UCLA: Statistical Consulting Group. How can I estimate the standard error of transformed regression parameters in R using the delta method? Accessed May 20^th^ 2021. <https://stats.idre.ucla.edu/r/faq/how-can-i-estimate-the-standard-error-of-transformed-regression-parameters-in-r-using-the-delta-method>

**Supplementary Figure S1 - Number of certified deaths in each state and territory 2015-2020**

**
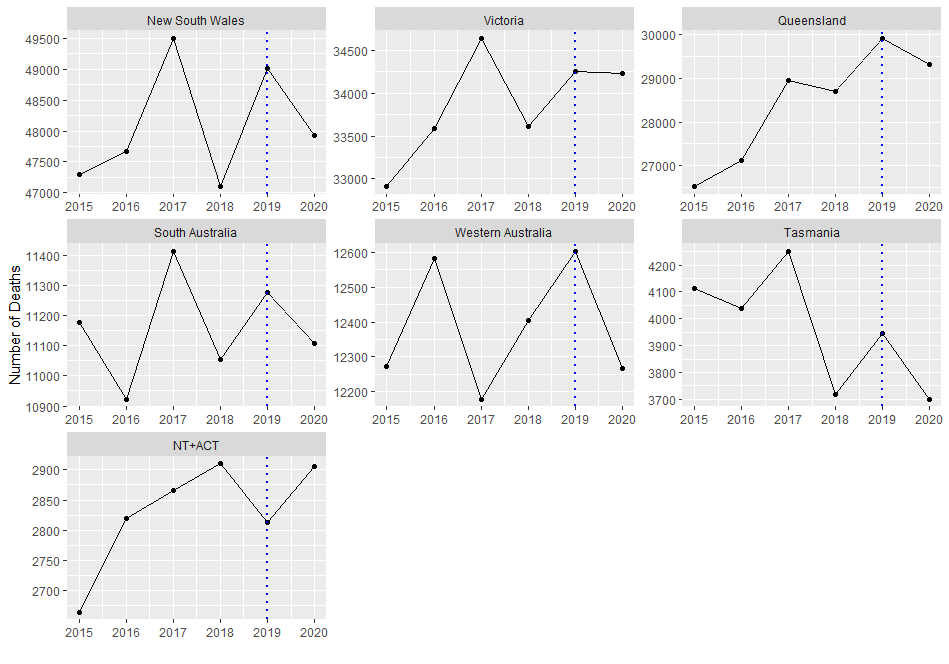
**

**Supplementary Table S1 – Week with peak number of certified deaths in each state and territory**

| **State** | **Phase (2015-19)** | **Phase (2020)** | **Shift (95% CI)** |
| --- | --- | --- | --- |
| **NSW** | 31 | 28 | -3 (-6 to 0) |
| **Victoria** | 31 | 30 | -1 (-4 to 1) |
| **Queensland** | 31 | 24 | -6 (-14 to 1) |
| **South Australia** | 32 | 28 | -4 (-11 to 3) |
| **Western Australia** | 32 | 30 | -2 (-8 to 4) |
| **Tasmania** | 33 | 40 | 7 (0 to 15) |
| **Northern Territory + ACT** | 30 | 30 | 0 (NA^#^) |

^#^Fitted model contained no seasonal interaction

**Supplementary Table S2 – Week with peak number of certified deaths by age and sex**

| **Sex** | **Age** | **Phase (2015-19)** | **Phase (2020)** | **Shift (95% CI)** |
| --- | --- | --- | --- | --- |
| **Male** | **0-44** | 30 | 14 | -16 (-26 to -7) |
|  | **45-64** | 30 | 30 | 0 (NA^#^) |
|  | **65-74** | 30 | 30 | 0 (NA^#^) |
|  | **75-84** | 31 | 28 | -3 (-8 to 1) |
|  | **85+** | 31 | 29 | -2 (-4 to 0) |
| **Female** | **0-44** | 31 | 31 | 0 (NA^#^) |
|  | **45-64** | 31 | 15 | -16 (-29 to -3) |
|  | **65-74** | 32 | 20 | -12 (-20 to -4) |
|  | **75-84** | 31 | 20 | -2 (-7 to 3) |
|  | **85+** | 32 | 30 | -1 (-4 to 2) |

^#^Fitted model contained no seasonal interaction

**Supplementary Table S3 – Week with peak number of certified deaths by cause of death**

| **Cause** | **Phase (2015-19)** | **Phase (2020)** | **Shift (95% CI)** |
| --- | --- | --- | --- |
| **Total Respiratory** | 33 | 19 | -14 (-22 to -5) |
| **Influenza & Pneumonia** | 34 | 17 | -17 (-21 to -12) |
| **Pneumonia** | 33 | 18 | -15 (-18 to -12) |
| **Chronic Respiratory** | 33 | NA^&^ | NA^&^ |
| **Cancer** | 23 | 23 | 0 (NA^#^) |
| **Ischaemic Heart Disease** | 31 | 30 | 0 (-3 to 2) |
| **Cerebrovascular Disease** | 30 | 30 | 0 (NA^#^) |
| **Dementia** | 30 | 22 | -8 (-12 to -3) |
| **Diabetes** | 30 | 27 | -3 (-8 to 1) |
| **All causes** | 31 | 26 | -5 (-8 to -1) |

^#^Fitted model contained no seasonal interaction

^&^ Seasonal effect almost entirely eliminated, numerical estimation of phase highly unstable
